# Quick guide on radiology image pre-processing for deep learning applications in prostate cancer research

**DOI:** 10.1117/1.JMI.8.1.010901

**Published:** 2021-01-06

**Authors:** Samira Masoudi, Stephanie A. Harmon, Sherif Mehralivand, Stephanie M. Walker, Harish Raviprakash, Ulas Bagci, Peter L. Choyke, Baris Turkbey

**Affiliations:** a National Cancer Institute, National Institutes of Health, Molecular Imaging Branch, Bethesda, Maryland, United States; b National Institutes of Health, Department of Radiology and Imaging Sciences, Bethesda, Maryland, United States; c University of Central Florida, Orlando, Florida, United States

**Keywords:** deep learning, medical images, image pre-processing, prostate cancer research

## Abstract

**Purpose**: Deep learning has achieved major breakthroughs during the past decade in almost every field. There are plenty of publicly available algorithms, each designed to address a different task of computer vision in general. However, most of these algorithms cannot be directly applied to images in the medical domain. Herein, we are focused on the required preprocessing steps that should be applied to medical images prior to deep neural networks.

**Approach:** To be able to employ the publicly available algorithms for clinical purposes, we must make a meaningful pixel/voxel representation from medical images which facilitates the learning process. Based on the ultimate goal expected from an algorithm (classification, detection, or segmentation), one may infer the required pre-processing steps that can ideally improve the performance of that algorithm. Required pre-processing steps for computed tomography (CT) and magnetic resonance (MR) images in their correct order are discussed in detail. We further supported our discussion by relevant experiments to investigate the efficiency of the listed preprocessing steps.

**Results:** Our experiments confirmed how using appropriate image pre-processing in the right order can improve the performance of deep neural networks in terms of better classification and segmentation.

**Conclusions:** This work investigates the appropriate pre-processing steps for CT and MR images of prostate cancer patients, supported by several experiments that can be useful for educating those new to the field (https://github.com/NIH-MIP/Radiology_Image_Preprocessing_for_Deep_Learning).

## 1 Introduction

In recent years, artificial intelligence (AI) has achieved substantial progress in medical imaging field where clinical decisions often rely on imaging data, e.g., radiology, pathology, dermatology, and ophthalmology.^1^ Within radiology, AI has shown promising results in quantitative interpretation of certain radiological tasks such as classification (diagnosis), segmentation, and quantification (severity analysis). It is usually a time-intensive, error-prone, and non-reproducible procedure when a radiologist evaluates scans visually to report findings and make diagnostic decisions.^2^ Alternatively, AI algorithms outperform the conventional qualitative approaches with faster pattern recognition, quantitative assessments, and improved reproducibility.^1^^–^^4^ More specifically, the advent of deep neural networks along with the recent extensive computational capacity enabled AI to stand out by learning complex nonlinear relationships in complicated radiology problems. As a result, deep learning-based AI has met and even surpassed human-level performance in certain tasks. However, training these usually large models, requires massive amounts of data, which can be limited in medical imaging applications due to the concerns over data privacy as well as the paucity of annotation (labels) in supervised learning. With a continuing trend for developing universal data anonymization protocols in addition to open data sharing policies, larger clinical datasets have started to become available. Thus, training on massive dataset composed of different sources (institutions with different scanners, image qualities, and standards) is an ongoing potential pathway. Nonetheless, in cases with inevitable limited data, possible solutions are to use simpler designs or to employ transfer learning strategies based on giant datasets of clinical or natural images.^5^ Either scenario, training on a big dataset or adopting transfer learning, are only two other important reasons that resonate the demand for a series of preliminary pre-processing steps prior to training; however, the nature of radiology images intrinsically necessities the preprocessing phase by itself.

Radiology images are acquired in a different way from natural pictures. Distinctive features of radiology images in each modality [i.e., computed tomography (CT) and magnetic resonance imaging (MRI)] are directly correlated with technical parameters used to generate these images. The detected (measured) signal within a scanner constitute the raw data that is reconstructed into an image in digital imaging and communications in medicine (DICOM) format as a standard in clinical medicine.^6^ DICOM files contain “metadata” in addition to the “pixel data,” which consists of image acquisition parameters, scanner information, and individual patient identification data. For clinical assessment, radiologists usually import this information as an image using the “picture archiving and communication system” (PACS).^7^ In some of their modality-specific and organ-specific workflows to fulfill a certain task, radiologists practice further adjustments to images often provided through a variety of third-party software programs embedded in PACS. These likely image modifications should be imitated as potential image pre-processing steps before training an AI system.

In the event that a massive dataset is built upon the data obtained from different patients, scanners, or multiple institutions, there are usually slight variations in the image quality, field of view, and resolution, which should be taken into consideration through a few pre-processing steps to create an integrated dataset.

In case of transfer learning, a model usually pre-trained on natural images is fine-tuned using radiology images. Natural images are obtained, stored, and loaded into memory with globally meaningful range of intensity values. However, based on the setting, clinical images are often acquired in multiple different ways with certain interpretation for their intensity values. Thus, to enable a robust transfer learning from the natural images into a dataset of radiology images, it is beneficial to apply several pre-processing steps.

Different pre-processing steps can also be viewed as a quality check for radiology images, which is beyond the embedded image quality filters at the scanner-level. To be more accurate, what is described in this paper is called post-processing according to medical physicists, and pre-processing according to image analysts. With that in mind, attaining a desired level of image quality in the training dataset can improve the succeeding quantification with deep learning.^6^

The nature of the pre-processing procedure strongly depends on the specific aim of the following processing algorithm and the image type. In practice, medical images can be provided in either formats of DICOM, analyze, Nifti, and Minc. Herein, our codes are based on the standard DICOM format;^6^^,^^8^ however, they can all be easily extended to any other format supported by the SimpleITK library.^9^ We also restrict our discussion to core pre-processing steps for anatomical MR and CT images with the aim of classification-, detection-, or segmentation-based applications using deep neural networks utilizing our institutional dataset of prostate cancer patients. Similar pre-processing methods can be extended to other imaging modalities or other medical research projects. Hence, we leave the choice of methods to users, with emphasis on the particular order that these methods should be applied to minimize the “uncertainties” coming from the data and enhance the performance of deep learning methods.^10^

### 1.1 What Do Medical Images Tell Us?

**Fig. 1:**
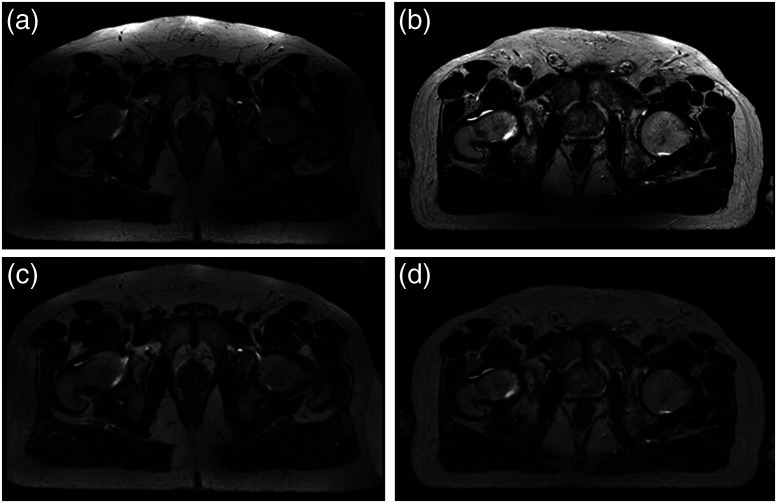
(a) and (b) represent the MR images of the same anatomical slice in two different patients obtained within the same scanner, where inhomogeneity and intensity non-standardness are very well demonstrated. (c) and (d) show the same slices after bias field correction.

The clinical goal of medical imaging is to provide anatomical and functional information. The most common modalities include CT, x-ray imaging, MRI, ultrasound, and positron emission tomography (PET). Each modality undergoes certain acquisition conditions that are essential for quantitative assessment. CT scans are acquired by measuring x-ray attenuation through a rotating energy source that produces cross-sectional images. CT imaging provides anatomical information due to differences in tissue composition (i.e., density). The nature of CT acquisition results in a quantitative measurement of tissue density relative to water, known as Hounsfield unit (HU). Voxel HU values in CT images are largely considered reproducible with slight differences across different scanners and patients following the standard temperature and pressure specifications. While CT provides strong contrast of major anatomical landmarks, it is not the preferred modality in some clinical settings due to ionizing radiation and its limited soft-tissue contrast. Instead MR imaging provides excellent resolution and contrast of soft-tissue components. MR images represent the radio-frequency energy released after re-alignment of the protons in the presence of a strong magnetic field. Since MR acquisition results in voxel values obtained relative to each other, these images are subject to significant variations even when the same scanner is used to scan the same patient or the same organ (Fig. 1).

## 2 Methods

**Fig. 2:**
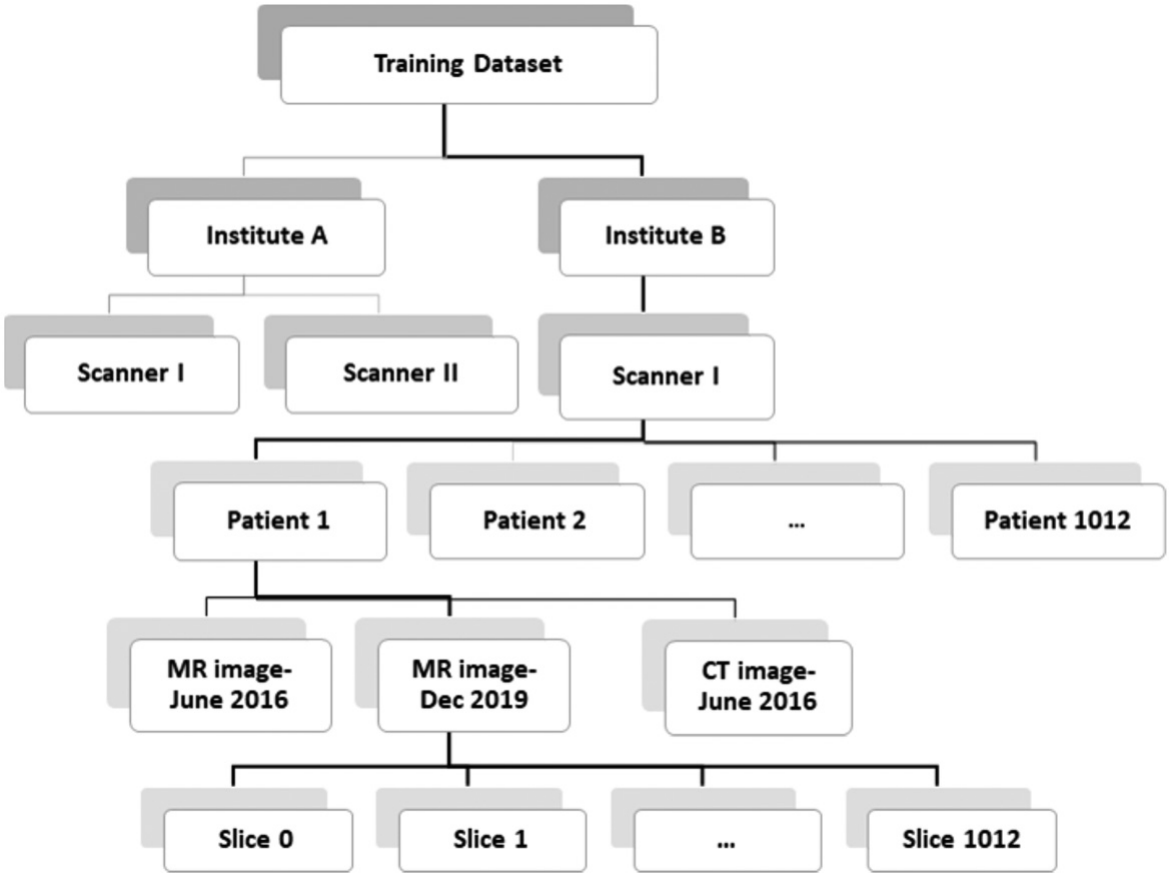
Depending on the ultimate task of deep neural network, pre-processing is performed at a certain level of a dataset employing the respective statistics.

In this section, we go through the details of pre-processing steps for CT and MR images, respectively. Considering the deep networks’ task, the pre-processing may need to be applied at a certain level. For instance, data normalization may be performed using the image statistics at the slice-level (each single slice within an image), image-level, patient-level, scanner-level, institution-level, or an overall training data-level (Fig. 2). It is common to use the patient-level pre-processing in most studies, but there may be applications for which other methods are better suited.

### 2.1 Pre-Processing the CT Images

In case of CT images, potential sequence of pre-processing steps may be listed in order as denoising, interpolation, registration, organ windowing followed by normalization, and potentially zero-padding to improve the quality of training for deep learning algorithms.

#### 2.1.1 CT data denoising

**Fig. 3:**
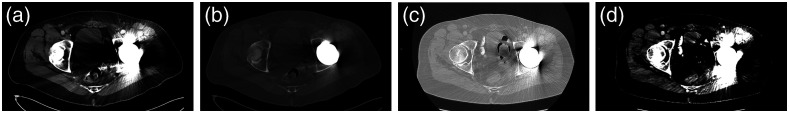
Images show left hip prosthesis related diffuse streak artifacts obscuring the CT image quality after (a) soft tissue; (b) bone; (c) lung; and (d) liver windows.

There are several sources of disturbance in CT images, which mainly include beam hardening, patient movements, scanner malfunction, low resolution, intrinsic low dose radiation, and metal implants (Fig. 3). Each of these disturbances is addressed individually in the literature.^11^^,^^12^ Generally, images can be denoised within two domains: (i) spatial and (ii) frequency, which are comprehensively discussed in the literature.^13^

#### 2.1.2 CT data interpolation

As it is usually the case for object detection and segmentation, there is a preference to have equal physical spacing for the input images. Maintaining the same resolution is desired to avoid center-specific or reconstruction-dependent findings. Images are usually interpolated in the xy plane and/or in z direction based on the desired physical spacing or ultimate number of the voxels. Function *ResampleImageFilter()* from the SimpleITK library performs this task with different interpolation methods. Cubic-spline and B-spline are generally better convolving functions to resample images as they perform close to an ideal low-pass filter.^14^ An inappropriate resampling step can negatively affect the subsequent registration and actual processing, such as degrading the resolution below the anticipated size of the detectable objects.^15^

#### 2.1.3 Registration of CT Data

**Fig. 4:**
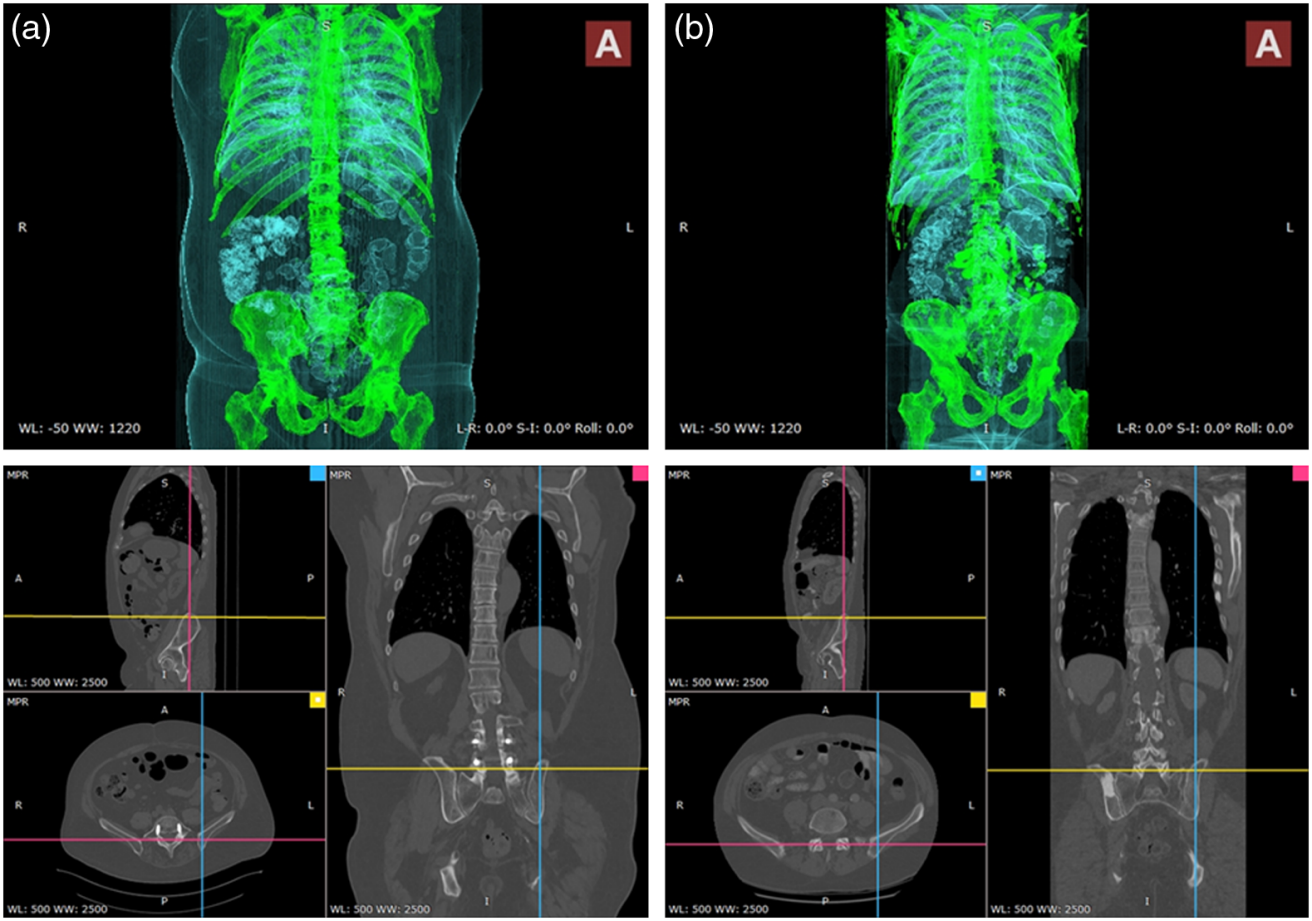
Registered field of view in CT data of patients A and B (Appendix A1).

Image registration implies a spatial transformation to align an area of interest in a floating image with a reference image. Medical image registration in radiology is interpreted in two different ways: (i) slice-level registration in an image due to patient movements during the scan and (ii) image-level registration to have comparbaly aligned images in a training dataset. The first registration type may be required only in certain CT images that are the product of slow scanning, prior to denoising and interpolation. The latter type of image registration can ease the learning process of space-variant features for classification or segmentation using conventional machine learning tools. We emphasize that registration as pre-processing is not as critical for object recognition based on deep learning as it used to be for conventional methods. Deep learning algorithms can be trained to learn features invariant to affine transformation or even different viewpoints and illuminations.^16^ Yet, to facilitate training in complicated problems, registration may prove useful. In addition, there could be scenarios demanding registered images as the input for deep neural networks. For instance, the quality of synthetic CT generation is negatively affected by poorly registered pairs of MR and CT scans in training.^17^ Figure 4 shows 3D CT images of two patients captured within the same scanner where we care to have registered anatomical field of view as the input. Limiting the field of view for various purposes during the image acquisition, data curation, and through image pre-processing can improve the processing results in terms of fewer false positives in general. There exists a wide spectrum of methods from simple thresholding to segmentation for image registration in the literature which are beyond the scope of this work.^18^^,^^19^

#### 2.1.4 Windowing in CT data

As mentioned earlier, CT images are originally acquired in 12- or 16-bit DICOM format to present measures of tissue density in HU which are integer numbers within an interval of (−1024  Hu, 4000 Hu). These values may change to real numbers after potential denoising, interpolation, and registration. There is a limit to direct presentation of these values as an image to a radiologist. According to perception literature, the human eye can only distinguish 100 shades of gray simultaneously while it can discriminate several thousand colors.^20^ Thus, for radiologists to be able to parse the complex images both faster and easier, one can incorporate color in clinical images or use image windowing to increase the contrast across a region of interest. In practice, the latter is commonly used where CT image values are clipped around a certain band of HU values for each organ, following unique standards from a texture-based dictionary described in Table 1. Obviously, computers are not limited by such a restriction, so windowing is not necessary if one is using the l6-bit images for training although they are not always supported by all deep learning libraries designed for 8-bit portable network graphics (PNG) or joint photographic experts group (JPEG) images.^21^ We believe storing images using l6-bit (either uintl6 or float numbers) could improve the results. To demonstrate this principle, we designed an experiment to explore the effect of windowing and the format of image storage on the validation accuracy of a visual geometry group-16 (VGG-16) binary bone lesion classification (benign versus malignant). The pre-trained VGG-16 network on ImageNet was trained and evaluated using the lesion patches extracted from staging CT images of prostate cancer patients. Further details about this experiment are provided in Appendix B1. The obtained results are shown in Table 2 which vary over a limited interval of (76.99%±0.8%). One can observe that bone windowing certainly improved the accuracy while keeping data in float format led to the best comparative performance. Hence, if not limited by format, float and uint16 are generally preferred over the 8-bit images. In case of the 8-bit images, CT windowing is generally advised to avoid a non-revertible compression of visual details, where CT data are clipped to fit in an interval of $W_{L}\pm W_{W}/2$ (where $W_{L}$ and $W_{W}$ are the window level/center and window width, respectively, following Table 1). Otherwise, it is a good idea to perform CT windowing within a wide interval of (−1350  Hu, 2000 Hu) to remove the extreme values caused by metal artifacts as shown in Fig. 3(b).

**Table 1:** Average Intensity Intervals in CT data that belong to different organs in four major areas of the body.^20^

Region	Organ	Intensity interval
Head and neck	Brain	(40±80/2)
	Subdural	(75±430/2)
	Stroke	(36±24/2)
	Temporal bones	(600±280/2)
	Soft tissues	(375±40/2)
Chest	Lungs	(−600±1500/2)
	Mediastinum	(50±350/2)
Abdomen	Soft tissue	(50±400/2)
	Liver	(30±150/2)
Spine	Soft tissue	(50±250/2)
	Bone	(400±1800/2)

**Table 2:** Evaluating the effect of windowing and storage on binary bone lesion classification of CT images in terms of validation accuracy. Bolded values indicate the improved results.

Windowing interval	Uint8 (%)	Uint16 (%)	Float32 (%)
(400±2000/2)	76.50	77.34	**78.17**
(0±4000/2)	75.63	**77.38**	76.90

#### 2.1.5 Normalization of CT data

To stretch or squeeze the CT data intensity values so that they efficiently fit into the provided range of input images (8 or 16 bits) monotonically, we simply use a linear transformation, enforcing two critical points (smallest and largest values) to be mapped to (0, 255) or (0, 65,535) respectively. Usually, we perform normalization at an institution-level or dataset level (utilizing the minimum and maximum pixel values among all the patients). A simple rule of thumb for implementing this is to use the windowing cut-off values of $W_{L}\pm W_{W}/2$ as the image extremes. Alternatively, one may normalize CT images at a patient-level, or even a slice-level for certain applications.

### 2.2 Pre-Processing the MR Images

While MR imaging is advantageous due to its superior soft-tissue contrast and non-ionizing radiation, these images are usually more challenging to be displayed or analyzed due to the lack of a standard image intensity scale. Additionally, MR imaging may be degraded by artifacts arising from different sources that should be considered prior to any processing. Inhomogeneity, motion and scanner-specific variations are among the major artifacts seen with the MRI. Figures 1(a) and 1(b) show how one similar anatomical slice in two different patients, obtained using the same scanner, have totally different intensity values. More importantly, Fig. 1 shows how the intensity values across the same organ within the same patient [for instance fat tissue in Fig 1(a)] vary in different locations. Potential steps to prepare MR images for further processing respectively include: denoising, bias field correction, registration, and standardization.^22^

#### 2.2.1 Denoising of MR data

Traditionally, denoising was an inevitable step to pre-process contaminated images. However, this phase is usually embedded within the current MR scanners, making acquired MR images to rarely suffer from direct noise distortion. In practice, additive Gaussian, Rician, and Speckle noise, respiratory and body movements and aliasing may still be major sources of contaminating noise in these images that should be addressed prior to any diagnosis. Motion can be avoided through several imaging protocols in this regard. There is also a long history of conventional methods proposed in the literature for motion artifact removal in MR images.^23^^–^^35^ Reliability of the derived diagnosis can be degraded by noisy image, which challenges both radiologists and automated computer-aided systems. To avoid false interpretations, it is critical to identify and exclude such poor images prior to any algorithmic analysis or inspection by the radiologists.

#### 2.2.2 MR bias field correction

**Fig. 5:**
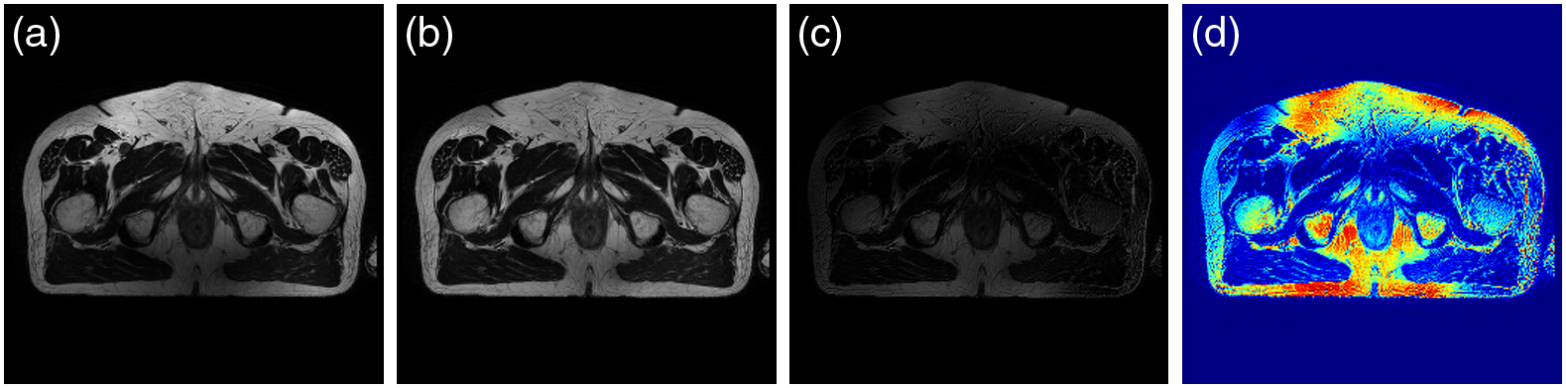
(a) and (b) represent the same MR slice with Bias field and after bias removal. (c) and (d) are the absolute difference and heat map of the respective bias field.

“Bias field” distortion is a low-frequency non-uniformity that is present in the MR data, causing the MR intensity values to vary across the images obtained from the same scanner, same patient, and even for the same tissue.^36^ Figure 5(a) shows this effect in an MR image obtained from the pelvic area. It is critical to perform bias correction on MR images prior to any registration.^36^^,^^37^ The accuracy of image registration not only depends on spatial and geometric similarity but also on the similarity of the intensity values of the same tissue across different images. There are segmentation-based methods that estimate the bias field as an image parameter using expectation–maximization (EM) algorithm.^38^^–^^40^ Other approaches are involved with image features instead, where N-4 ITK is the most commonly used method among them.^9^^,^^41^ This approach was first proposed by Tustison et al.,^41^ and is available in SimpleITK library. We have shown the results of bias field removal in Figs. 5(c), and 5(d).

#### 2.2.3 Registration of MR data

Similar to CT, MR scanning may leads to mis-aligned images that require image registration. In fact, since MR scanning is much slower than CT, MR slices within the same image sequence are more likely to be offset from one another. While slice-level registration is required when processing 3D images within one channel, image-level registration becomes also essential while processing various modalities all together.^42^^,^^43^ A critical example could be applications where image fusion or domain adaptation is aimed between MR and CT images obtained using different fields of view during separate retrospective studies.^16^

#### 2.2.4 MR data standardization

**Fig. 6:**
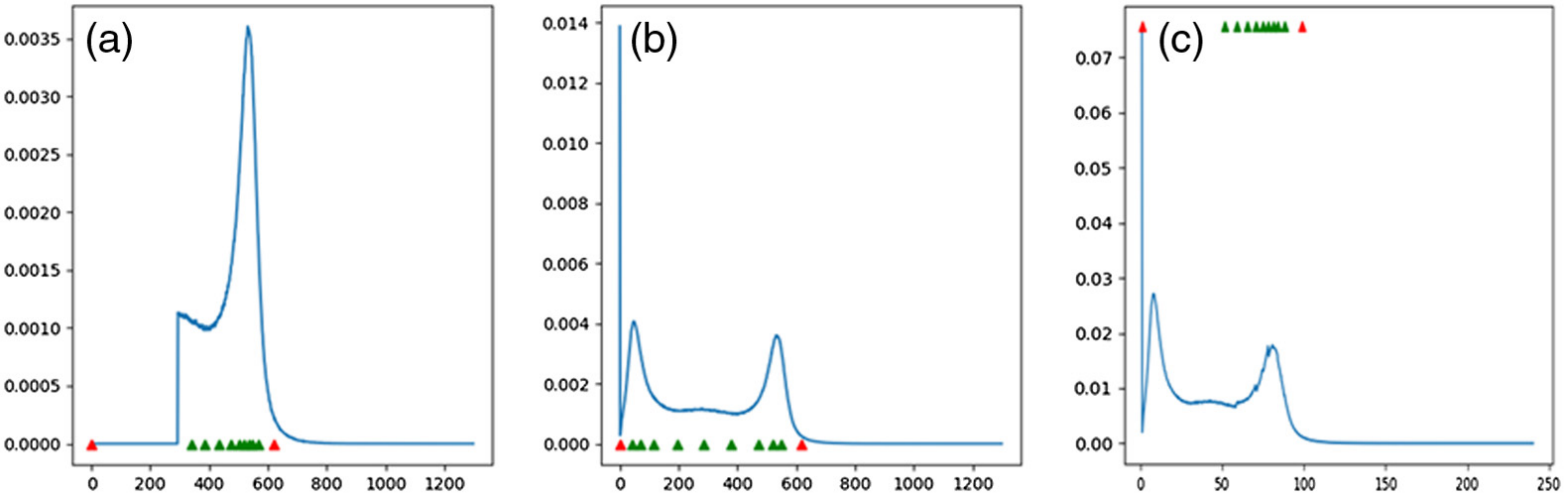
(a), (b), and (c) represent the foreground PDF with standard landmarks, original PDF, and the resulted mapped PDF associated with Fig. 7.

**Fig. 7:**
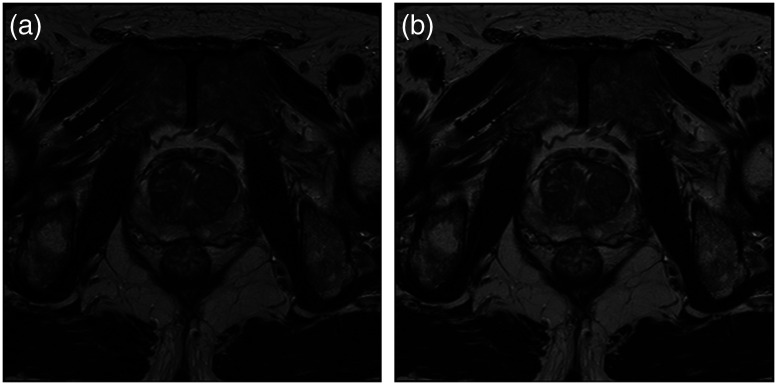
(a) and (b) represent the same MR slice before and after Nyul preprocessing using 10 green landmarks in Fig. 6.

To have comparable intensity values in MR images, we must enforce image standardization so that for the same MR protocol within the same body region, a particular intensity value represents a certain tissue across all different slices and patients. Many studies in the literature use the standardization method proposed by Nyul and Udupa^44^ to alleviate this problem. However, prior to Nyul pre-processing, we may: (i) shift the intensity values into a positive range of numbers, and (ii) use the probability distribution function (PDF) of the image intensity values to cut off the upper or lower narrow tails of the histogram. The latter can potentially help to remove very rare incidents of a certain intensity values caused by noise. Afterward, images can be standardized through a two-step Nyul pre-processing method which illustration is shown in Figs. 6 and 7. The first step implies the learning phase where parameters of the standardizing transformation are learned from images in the training set. Next is the transforming phase where the intensity values of all images (training, validation, and validation) are mapped into new values using the standardizing transformation computed during the learning phase. Doing so, the PDF of each image would match a standard PDF. It is worthy to note that such standard PDF for MR data is approximated by a bi-modal distribution where the first bump usually represents the background. In practice, the Nyul method is focused on mapping the foreground or the second bump in histogram using a certain number of the landmarks [10 landmarks in Fig. 6(c)].

### 2.3 Demonstration

Finally, to demonstrate the efficacy of the aforementioned pre-processing methods in improving the performance of deep neural network algorithms, we performed four experiments with the aim of classification and segmentation in CT and MR images, i.e., two experiments for each image modality. These tasks can be described as (1) binary bone lesion classification (benign versus malignant) of 2-D lesion patches extracted from the CT data of proven prostate cancer patients; (2) binary 2-D slice-level classification (class 0 implies containing lesion of at most PIRADS 1,2 and class 1 describes slices with higher risk lesions PIRADS 3,4,5) of T2-weighted MR scans of prostate cancer patients from ProstateX dataset;^45^ (3) visceral fat segmentation of 2-D slices from abdominal CT-scans; and (4) whole prostate segmentation in 2-D slices of T2-weighted MR images from ProstateX dataset.^45^ For classification and segmentation purposes, we used two popular algorithms, VGG-16, and U-net, respectively. Our classifiers are trained taking advantage of transfer learning on the ImageNet, while U-net was trained from scratch in each case. More details are provided in Appendix B.

**Fig. 8:**
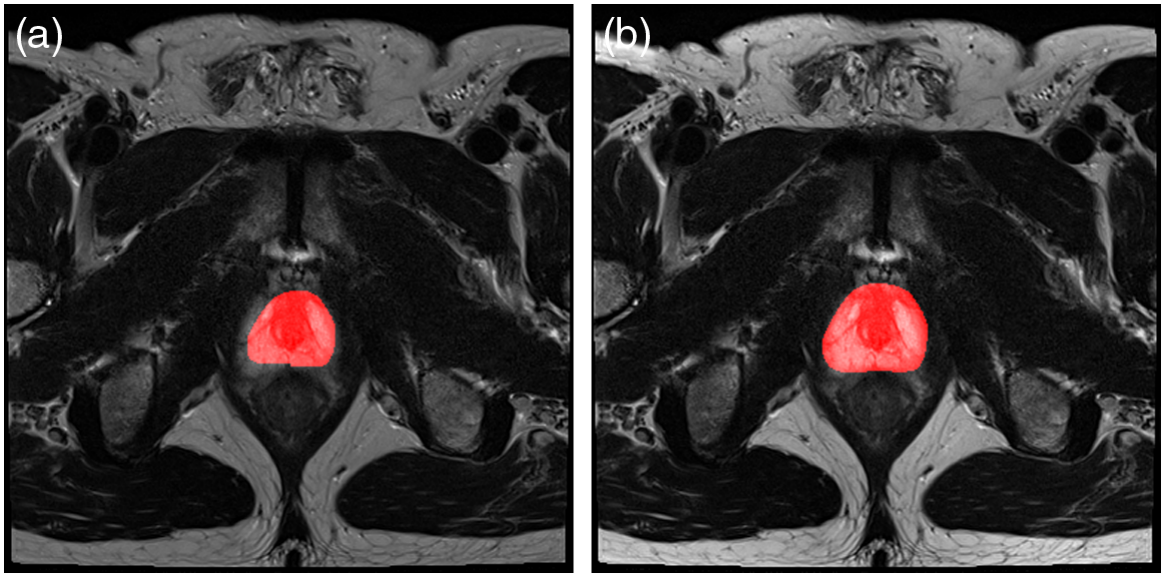
An example of whole prostate Segmentation (a) without pre-processing (mean absolute error of 28.4%) and (b) with pre-processing (mean absolute error of 4.3%).

The results with and without the pre-processing are shown in Table 3 that confirm the necessity of appropriate pre-processing steps in the suggested order. While classification accuracy in both patch-level (CT) and slice-level (MRI) has increased significantly, the profound effect of pre-processing on segmentation performance is acutely presented both in terms of mean absolute error and dice score. The results in Table 3 also signify the demand for pre-processing in MRI images compared to CT images. Figure 8 shows an example of T2-MR slice where pre-processing could prevent under-segmentation of the whole prostate by U-net.

**Table 3:** Evaluating the effect of pre-processing on classification and Segmentation tasks in terms of validation accuracy. Bolded values indicate the improved results.

Task	Image type	Criteria	With pre-processing (%)	No pre-processing (%)
Classification	MR	Accuracy	**73.30**	68.74
	CT	Accuracy	**82.28**	77.72
Segmentation	MR	Mean Abs. Err.	**2.73**	47.64
		Dice	**98.64**	81.74
	CT	Mean Abs. Err.	**3.68**	19.99
		Dice	**98.25**	95.25

## 3 Discussion and Conclusions

Certainly, one major area in which AI is ascendant is the field of radiology. Conventionally, radiologists have exploited their scientific knowledge and visual ability to interpret medical images for diagnostic decisions. While such tasks are laborious and time-intensive, the results are excessively error-prone.^2^ Current shortcomings necessitate an alternative solution. With the advent of deep neural networks, certain radiologist’s tasks can be assigned to pre-trained machines instead. However, such a revolutionary substitution toward automation highly depends on how radiologists can understand the potentials of these algorithms. Currently, visual computation tasks in radiology addressed with deep neural networks mainly include but are not limited to detection, classification, and segmentation. In due time, it is critical for every radiologist to learn to: (1) define a problem with regards to the capabilities of deep learning algorithms; (2) collect and label the relevant data consistent with the algorithm to address the problem; (3) perform the necessary pre- processing steps to prepare the input data; and (4) choose an appropriate algorithm which will be trained on the provided data to solve the problem. There is a lack of thorough references available to educate radiologists on pre-processing as the first step. It is necessary for both radiologists and data scientists to understand these preprocessing steps so they can work effectively together to create ideal solutions. We hope this compilation of codes from the public domain will be useful.

## 4 Appendix A: Registration Details

### 4.1 Registration of CT data

As it can be seen in Fig. 3, both patients in Fig. 3 are adjusted to have the same axial view in 512×512 axial plane which results in different x and y spacing. On the other hand, with similar z-spacing (slice thickness) of 1 mm, images (a) and (b) are turn out to have a different number of slices, 663 and 778, respectively, while presenting a similar view in the z direction. With all these considerations, due to slight differences in measurement specification and organ-specific spatial organization of the patients, (x,y,z) physical spacing can not be used as a reference for registration.

## 5 Appendix B: Implementation Details

### 5.1 Bone lesion classification in CT scans

A dataset of 2879 annotated bone lesions from CT scans of 114 patients diagnosed with prostate cancer from the National Cancer Institute, National Institutes of Health was used for this experiment. We utilized 85 cases with 2224 lesions to train a pre-trained VGG-16 classifier and validated our results on 29 cases with 655 lesions. We used lesion annotations in terms of their labels of either being benign or malignant to define the classification task. Through 80 epochs of training, we used ADAM optimizer to minimize our binary cross-entropy loss function, initiated from the base learning rate of 3e−3 with 0.2 iterative drop, in learning rate in every 5 epochs.

### 5.2 PI-RADS classification of T2-weighted MR images from ProstateX dataset

We used whole prostate segmentation of 347 patients from publicly available Prostate X dataset including the T2-weighted MR scans along with internal annotation for lesions using 5-scale PI-RADS categorizations. We extracted 2D slices (N=2866) from T2-weighted MR images where each 2D slice is assigned a class 0 (N=1394) or 1 (N=1472). In our dataset, class 0 implies that lesions of at most PI-RADS 2 are contained within the 2D slice while 2D slices with class 1 carry higher risk lesions with at least PI-RADS 3. The data were split into a training cohort of 300 patients and 47 patients for evaluation. We defined our loss function based on categorical cross-entropy, minimized it during 60 epochs of training, using ADAM optimizer with a base learning rate of 1e−3 along with 0.2 iterative drop in every 7 epochs.

### 5.3 Visceral fat segmentation in CT-scans of abdominal area

We used CT scans (from the abdomen region) of 131 patients obtained from multiple centers (Mayo clinic) with their respective visceral fat labels for segmentation. We randomly split these patients into two groups of train (N=115) and validation (N=16) to respectively train and validate our U Net-based segmentation network. To facilitate the training for the complicated task of visceral fat segmentation, we extended the limited number of the annotated CT slices, using augmentation methods such as image translation (width and height), reflection (horizontal flip), and zooming to increase (by 4 folds) our set of training images. Defining a pixel-wise binary cross-entropy as our loss function, we used SGD to increase the generalizability of our model with the base learning rate of 5e−4 and iterative decrease in learning rate. Through 100 epochs for training, we maintained 8 multiples of the learning rate in every 12 epochs.

### 5.4 Whole prostate segmentation on T2-weighted MR images from ProstateX dataset

We used 99 cases of publicly available T2-weighted MR scans from Radboud University Medical Center in Nijmegen, Netherlands, with internal annotation for whole prostate segmentation. We extracted 2D slices (N=2866) from T2-weighted MR images. The data were split into a training cohort of 85 patients for training and 14 patients for evaluation. We forced training by 100 epochs for minimization of pixel-wise binary cross-entropy using SGD optimizer with base learning rate of 5e−4 and 0.2 iterative drop every 15 epochs.

## Code, Data, and Materials Availability

Supporting the research results reported in the manuscript, we provided the organized preprocessing codes for MR and CT images at https://github.com/NIH-MIP/Radiology_Image_Preprocessing_for_Deep_Learning.
